# Culturally adapted body image program for Mexican university women: feasibility, acceptability, and cultural relevance in a Pilot RCT protocol

**DOI:** 10.3389/fpsyt.2025.1717786

**Published:** 2026-01-14

**Authors:** Eva M. Trujillo-ChiVacuán, Bertha Winterman-Hemilson, Elsie Y. Trujillo-Valdes, Anid Cortes-Morales, Emilio J. Compte

**Affiliations:** 1 Research Department, Comenzar de Nuevo AC, Monterrey, Mexico; 2 Tecnologico de Monterrey Escuela de Medicina y Ciencias de la Salud, Monterrey, Mexico; 3 Eating Behavior Research Center, School of Psychology, Universidad Adolfo Ibáñez, Santiago, Chile

**Keywords:** body dissatisfaction, body image program, cultural adaptation, eating disorders prevention, feasibility trial, Mexico, pilot-randomized controlled trial, university students

## Abstract

**Background:**

Eating disorders (EDs) and body dissatisfaction (BD) are common among young women in Mexico, yet the evaluation of culturally adapted prevention programs remains limited. Early work implementing a dissonance-based intervention with Mexican university students reported encouraging changes in BD and thin-ideal internalization. Building on this initial evidence, there is a clear need for a randomized design to examine the feasibility and implementation of culturally adapted prevention efforts in this context.

**Methods:**

This single-center, two-arm pilot RCT will recruit 30 female university students aged 18–25 years in Northeastern Mexico. Participants will be randomized 1:1 to the Body Image Program (BIP) or a waitlist control. Intervention groups will receive two 120-minute in-person sessions over consecutive weeks. Assessments will occur at baseline (week 0), post-intervention (week 2), and follow-up (week 6). Waitlist participants will be offered the BIP after the final follow-up. Primary outcomes are feasibility and acceptability (recruitment ≥70%, retention ≥80%, adherence ≥70%, satisfaction ≥4/5) and ED symptoms (ED-15). Secondary outcomes include BD (BSQ-8), body appreciation (BAS-2), social physique anxiety (SPAS-7), thin-ideal internalization (SATAQ-4), and appearance comparisons (PACS). Analyses will use linear mixed-effects models under an intention-to-treat framework, reporting standardized effect sizes with 95% confidence intervals.

**Conclusions:**

This protocol describes a pilot randomized trial of a culturally adapted body image program in a Mexican university setting. The study will provide feasibility data to guide a fully powered RCT and contribute to the development of culturally relevant prevention strategies in Latin America.

**Clinical Trial Registration:**

ClinicalTrials.gov, identifier: NCT07193043.

## Introduction

Eating disorders (EDs) and disordered eating behaviors are highly prevalent conditions with serious health and social consequences, including high psychiatric and medical morbidity, increased premature mortality (1) and substantial comorbidity with mood, anxiety, and substance-use disorders, as well as considerable social and economic costs (2, 3). Beyond clinical diagnoses, disordered eating behaviors such as restrictive dieting, binge eating, and unhealthy weight-control practices are also highly prevalent in community populations and represent important risk factors for the onset of full-syndrome EDs (4). Although cognitive-behavioral and dissonance-based prevention programs have shown consistent efficacy in reducing ED risk factors (4–6) most evidence derives from Western populations. This Eurocentric evidence base raises concerns about transferability to culturally diverse contexts, reinforcing calls to bridge cultural adaptation and implementation science to ensure both fidelity and contextual fit (7, 8).These concerns are central to current efforts to address inequities in ED prevention and to conceptualize interventions along a cultural adaptation continuum.

In Mexico, national epidemiological data illustrate the urgency of this issue. The Encuesta Nacional de Salud y Nutrición (National Health and Nutrition Survey), a large-scale population-based survey, reported a high prevalence of risky eating behaviors among adolescents, underscoring the early emergence of risk in this group and its relevance for public health in the country (9). These findings, in conjunction with international evidence of an increasing risk in young women (5, 10), underscore the need for preventive strategies that can mitigate risk factors before the onset of clinical disorders, particularly in adolescents and young adults. In Mexican universities, surveys suggest high rates of dieting, BD, and weight-control behaviors, reinforcing the urgency of prevention strategies tailored to young women in higher education settings. Studies of national burden show that between 1990 and 2021, the age-standardized disability-adjusted life years (DALYs), a composite measure of years of life lost and years lived with disability, for anorexia nervosa and bulimia nervosa increased more than 50% in México, denoting a substantial increase in the burden associated with these disorders (11). Global comparative data from the Global Burden of Diseases, Injuries, and Risk Factors Study 2019 study further highlight the rising burden of EDs worldwide, underscoring the need to contextualize these findings from Mexico within broader international trends (12).

Beyond prevalence, EDs are associated with a considerable social and economic burden, including high treatment costs, reduced productivity, and long-term disability (2, 3). Although such costs have not yet been systematically estimated in Latin America, the high prevalence of risky eating behaviors documented in national surveys (9) suggests a substantial public health impact in the region. This underscores the urgency of developing and evaluating preventive interventions that can be feasibly implemented in local contexts.

Eating disorders contribute substantially to the global burden of disease and are associated with impaired mental health and reduced quality of life (3). Although cost estimates are scarce in Latin America, indirect evidence suggests significant healthcare expenditures, productivity loss, and psychosocial disability associated with EDs (11, 13). Systematic reviews emphasize the lack of culturally adapted interventions in the region and highlight the urgency of developing preventive approaches in underrepresented populations (14).

Among preventive interventions, the Body Project stands out as one of the most extensively studied and efficacious programs. Developed as a dissonance-based intervention, it engages participants in verbal, written, and behavioral exercises that critique the thin-ideal standard of beauty, thereby reducing internalization of unrealistic appearance ideals. The program is typically delivered in small groups across two to four sessions and has demonstrated consistent efficacy in reducing BD, thin-ideal internalization, dieting, negative affect, and ED symptoms in adolescent and young adult women (4, 6). Meta-analytic evidence further supports its effectiveness and durability of effects (4). Other trials have also demonstrated sustained long-term effects, including a 3-year follow-up showing significant reductions in risk factors and symptoms (15). Adaptations have extended their reach to mixed-gender groups and community settings, with promising results (5, 10). Beyond efficacy, however, achieving population-level impact requires attention to delivery systems, including access, engagement, and sustainability, in addition to cultural and linguistic adaptations (8). This evidence base positions the Body Project as a leading model for ED prevention internationally.

The rationale for interventions targeting body image concerns is grounded in well-established theoretical frameworks. The sociocultural model of ED pathology (16) and the Tripartite Influence Model (17) both posit that sociocultural pressures and the internalization of appearance ideals contribute to BD, which in turn predicts disordered eating behaviors (18). The transdiagnostic cognitive-behavioral theory of EDs similarly highlights the central role of body image overvaluation in the onset and maintenance of symptoms (19). Together, these models provide a strong theoretical foundation for preventive interventions that aim to reduce the internalization of unrealistic beauty standards and BD.

In Latin America, efforts have been made to adapt and implement the Body Project to local contexts. Unikel-Santoncini et al. (20) conducted a quasi-experimental study with 40 Mexican female university students who participated in a two-session dissonance-based program. Results showed reductions in BD and thin-ideal internalization. However, the absence of a randomization and a control group limited the strength of the conclusions and underscored the need for more rigorous trials in the region. More recently, (21) documented the adaptation and large-scale implementation of the Body Image Program (BIP) across Mexico and Latin America. This work described the translation, cultural adaptation, and dissemination of BIP through a tiered training system that has prepared more than 700 facilitators in multiple countries, reaching schools, universities, companies, healthcare professionals, and community organizations. The study highlighted the feasibility of embedding weight stigma reduction into program delivery, the sustainability of implementation via business sponsorships, and the scalability achieved through a train-the-trainer model. These findings underscore the promise of culturally adapted prevention programs in the region but also make clear that evidence to date comes primarily from dissemination and implementation studies rather than rigorously controlled randomized trials (14).

In evaluating the impact of prevention programs, it is essential to capture not only changes in ED symptoms and BD but also related mechanisms and functional outcomes. Consistent with sociocultural and cognitive-behavioral models, thin-ideal internalization and appearance-based social comparisons are considered central risk factors for the development of EDs (16, 17). Appearance-related anxiety, such as concerns about being negatively judged by others, has also been identified as a relevant vulnerability factor (22). Beyond symptomatology, assessing psychosocial impairment provides valuable information on the extent to which ED-related difficulties interfere with daily functioning (3). Importantly, recent work has emphasized the relevance of positive indicators of body image, such as body appreciation (23), to complement traditional risk-focused measures. Including these outcomes allows for a comprehensive evaluation of both risk reduction and the promotion of protective factors in the context of ED prevention.

In choosing a design for preliminary evaluation, pilot randomized control trials (RCT) are generally considered preferable to uncontrolled or feasibility-only studies because they allow researchers to examine the integration of all trial components under conditions that closely approximate a definitive RCT. Unlike open trials, which cannot rule out alternative explanations for change, or feasibility studies that are often limited to assessing recruitment or acceptability, pilot RCTs provide an initial test of recruitment, retention, randomization, intervention delivery, and outcome assessment within the same framework. This makes them particularly valuable for informing the design and implementation of subsequent large-scale trials (24–27).

Taken together, these considerations point to the need for rigorous preliminary research on culturally adapted prevention programs in Latin America. Preliminary evidence from Mexico has demonstrated the feasibility and potential benefits of dissonance-based prevention programs (20, 21), yet the absence of randomized controlled trials in the region leaves a critical gap in the evidence base. The present study addresses this gap by reporting the pre-registration of the first pilot RCT of the Body Image Program (BIP) in Mexico, designed to evaluate feasibility, acceptability, and preliminary efficacy among female university students. Pilot studies serve as preparatory work for subsequent large-scale RCTs, primarily aimed at assessing feasibility and informing the design of future trials (25–28).

## Method

### Study design

**Figure 1:**
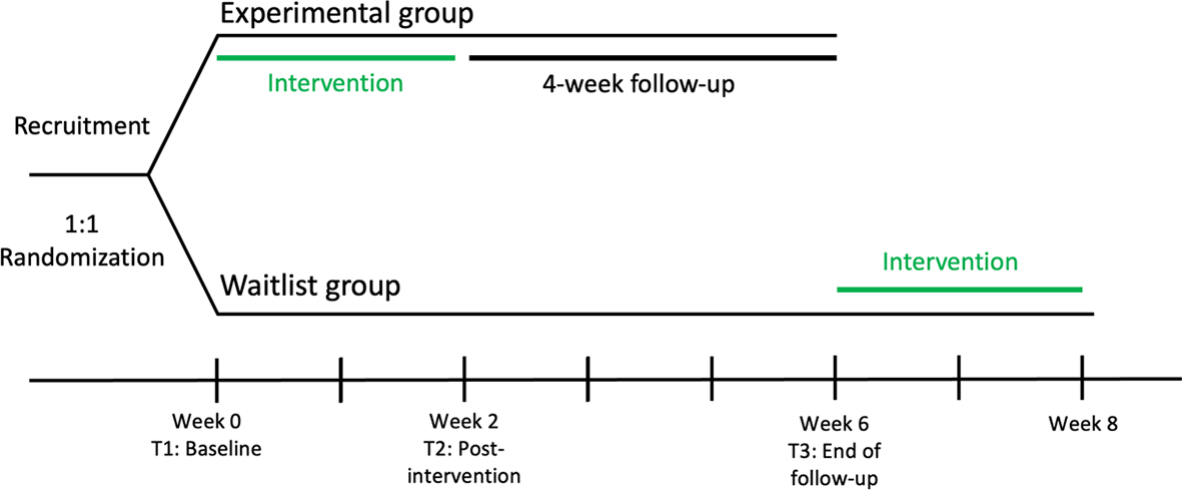
Study design and timeline of the pilot randomized controlled trial. Participants were randomized 1:1 to the experimental group or the waitlist control group. Both groups completed assessments at baseline (T1, week 0), post-intervention (T2, week 2), and follow-up (T3, week 6). The experimental group received the two-session BIP intervention during weeks 0–2, followed by a 4-week follow-up period. The waitlist group received no intervention during the study period but was offered the BIP between weeks 6 and 8 after completing T3. Data from the delayed intervention period were used only to document feasibility indicators.

This study is a two-arm pilot randomized controlled trial (RCT) designed to evaluate the feasibility and acceptability of the BIP for the prevention of eating disorders among female university students in northeastern Mexico. The trial was preregistered at ClinicalTrials.gov (Identifier: NCT07193043). Participants will be randomly assigned in a 1:1 ratio to either the intervention (BIP) or a waitlist control group. The intervention will be delivered in small groups using the culturally adapted version described by Trujillo-ChiVacuan et al. (21). Assessments will be conducted at three time points: baseline (T1, week 0), immediately post-intervention (T2, week 2), and at 4-week follow-up (T3, week 6). Participants in the waitlist control group will be offered the BIP after completing the T3 follow-up assessment, between weeks 6 and 8 (see **Figure 1** for an overview of the study design). No additional follow-up assessments are planned after the waitlist group receives the program. Data collected from this delayed intervention cohort will be used only to document feasibility indicators (e.g., participation and attendance), as the pilot is not powered to evaluate effectiveness in this group.

The choice of a pilot RCT design is consistent with recommendations in the methodological literature, which emphasize that pilot studies are preparatory versions of larger definitive trials, aimed primarily at evaluating feasibility processes such as recruitment, randomization, intervention delivery, and retention (25–28). Unlike feasibility studies, which focus on estimating parameters to inform sample size calculations, pilot RCTs test the integration of all study components under conditions that closely mirror those of a full trial (27). This design is therefore appropriate for determining the practicality and acceptability of implementing the BIP in this context, while also generating preliminary estimates of outcome variability to inform a future fully powered RCT. Consistent with this purpose, the short-term follow-up schedule (2 and 6 weeks) was intentionally selected to assess feasibility, retention, and early signals of change, which are the core aims of external pilot trials, with longer-term outcomes reserved for the subsequent definitive RCT. The protocol was developed in accordance with the SPIRIT 2013 guidelines (29).

### Participants and recruitment

Participants will be recruited from a single university in northeastern Mexico through classroom announcements, institutional mailing lists, social media, and campus posters. All study procedures will take place on campus. Because the BIP is adapted from the Body Project, which was originally developed for university settings, targeting female university students ensures consistency with the intervention’s theoretical and developmental context. Inclusion criteria: Female undergraduate students, aged 18–25 years, currently enrolled at participating universities in northeastern Mexico, with sufficient Spanish language proficiency to complete the intervention and assessments, and able to provide written informed consent. Exclusion criteria: Self-reported current diagnosis of a severe eating disorder requiring specialized treatment (i.e., a previous diagnosis given by a health professional that the participant considers currently needs specialized care), current engagement in intensive psychiatric or psychological treatment (i.e., weekly treatment with a clinician, participation in a structured program, or recent hospitalization), or insufficient availability to attend the two scheduled intervention sessions.

Participation will be voluntary. To support retention and acknowledge participants’ time and effort, all participants will receive a modest gift card after completing the T3 assessment. Compensation is identical for both groups, is not contingent on adherence or study outcomes, and does not constitute a study benefit.

### Intervention

Participants randomized to the intervention arm will receive the BIP, a culturally adapted, dissonance-based group intervention derived from the Body Project (21, 30). Cultural adaptations include (a) linguistic modifications for Mexican Spanish, (b) replacement of U.S.-centric appearance examples and media imagery with locally relevant content, (c) incorporation of sociocultural pressures specific to Latin American beauty ideals, such as the curvy-thin ideal and the normalization of appearance-based teasing, and (d) culturally relevant behavioral challenges that target real-life situations common among Mexican university students. Additionally, the intervention was renamed as the Body Image Program (BIP) to improve clarity and acceptability in Spanish-speaking contexts, while preserving the theoretical foundation and session structure of the original Body Project. These adaptations were developed and evaluated in prior BIP implementation studies in Mexico and Latin America (21), which demonstrated feasibility and acceptability, but did not include randomization or a control group. The program consists of two 120-minute group sessions, delivered once per week over two consecutive weeks, in small groups of 7–10 participants (maximum 10). When the intervention arm exceeds one group’s capacity, participants will be scheduled into two concurrent groups of approximately 7–10 each to preserve group processes and logistics. Sessions include verbal, written, and behavioral exercises to elicit cognitive dissonance, critically evaluate sociocultural appearance ideals, and foster more adaptive body-image perspectives. A session-by-session overview is provided in **Table 1**.

**Table 1:** Overview of the Body Image Program (BIP): objectives, core activities, and homework.

Session	Main objectives	Core activities	Homework assignments
1.	Introduce the program; establish voluntary commitment and group cohesion; identify and analyze sociocultural origins and costs of the thin-ideal; initiate a critical stance via dissonance.	Guided discussion on sociocultural pressures and personal/interpersonal/social costs of pursuing the thin ideal; written counter-ideal arguments to generate cognitive dissonance; introduction and in-session practice of the mirror exercise; assignment of a behavioral challenge to reinforce learning between sessions.	Daily practice of the mirror exercise; completion of the behavioral challenge introduced in session; reflective letter to a younger girl describing challenges and insights related to appearance pressures.
2.	Reinforce commitment; deepen critical evaluation of appearance ideals; practice resistance strategies; strengthen values and self-affirmation; program closure	Role-plays to resist appearance pressures; reflective writing (e.g., letter to a younger person), mirror reflection and group discussion of experiences; values list (“top ten”); self-affirmation exercises; discussion of group benefits and formal closure	Commitment task (e.g., self-affirmation, body activism, “10 best ideas” list, or reflective letter). Assignments to be submitted electronically within one week.

BIP consists of two weekly 2-hour group sessions facilitated by trained professionals. Materials were culturally adapted into Spanish as described in Trujillo-ChiVacuan et al. (21). Homework tasks are conceptualized as engagement tasks designed to reinforce session content, rather than as measures of adherence.

The intervention will be delivered by trained health professionals (Group Leaders) with prior experience in eating disorders, who have completed the standardized BIP training program and receive ongoing supervision to ensure adherence to the protocol. The standardized BIP training follows a train-the-trainer model and includes approximately 16 hours of experiential instruction combining theoretical foundations, practice-based demonstrations, and supervised mock sessions to ensure facilitator proficiency (21). Fidelity will be monitored through session checklists completed by Group Leaders and periodic supervision meetings with senior BIP trainers. If pre-session attrition reduces a group below seven participants, sessions may be rescheduled or participants reassigned to maintain the minimum group size. The program will be delivered in Spanish using culturally adapted materials developed in prior implementation studies in Mexico and Latin America (21).

Each session concludes with homework designed to reinforce dissonance-based learning. These tasks are considered part of participant engagement but will not be included in the definition of adherence, which is based on attendance to the two intervention sessions. After Session 1, participants receive structured tasks (e.g., a behavioral challenge, reflective writing, mirror exercise). At the end of Session 2, a commitment assignment is provided (e.g., self-affirmation or body-activism activity) intended to consolidate program principles during the weeks prior to follow-up. Participants are asked to complete this final assignment within one week of Session 2 and to submit it by email to the Group Leaders, ensuring engagement during the immediate post-intervention period.

Participants allocated to the waitlist control condition will complete the same assessment schedule as the intervention group (T1, T2, T3) but will not receive any intervention during the study period. After the final follow-up assessment (T3, week 6), they will be offered the BIP between weeks 6 and 8, in line with ethical considerations and the principle of post-trial access for control participants. This approach is consistent with the principles of the Declaration of Helsinki, which emphasize that participants in control conditions should not be denied access to potentially beneficial interventions once the main study assessments are completed (42).

### Facilitator training

Each session will be delivered by two trained Group Leaders, who are health professionals with prior experience working with eating disorders. All leaders must have completed the standardized BIP training program conducted by headmaster trainers. The training follows an experiential approach that integrates theoretical foundations with practice in both facilitator and participant roles, ensuring the development of technical competencies and a comprehensive understanding of group processes. Leaders also receive ongoing supervision during implementation, which supports fidelity to the intervention protocol, consistency across groups, and effective management of group dynamics.

### Outcomes and measures

Feasibility and acceptability outcomes will be assessed following guidance for pilot RCTs (24, 27). Recruitment will be indexed as the proportion of eligible students who consent and are randomized, with ≥70% considered acceptable. Retention will be measured as the percentage completing T2 and T3 assessments, with a target of at least 80%. Adherence will be defined primarily as attendance, categorized per participant as full (2/2 sessions), partial (1/2), or none (0/2), with ≥70% full adherence considered acceptable. Submission of the Session-2 commitment task within one week will be examined separately as an indicator of engagement, consistent with the preregistered protocol, and will be reported independently from adherence. Acceptability will be evaluated through a brief post-intervention survey at T2 on usefulness, clarity, and relevance, with favorable acceptability defined *a priori* as a mean rating ≥4 on a 5-point scale. Feasibility indicators will be summarized descriptively using percentages and 95% confidence intervals, without formal hypothesis testing.

The primary clinical outcome will be change in eating disorder symptoms, assessed with the Eating Disorder-15 (ED-15; 31), a 15-item self-report instrument that evaluates core symptoms over the past week. Items are rated on a 0–6 Likert scale and yield two subscales (Eating Concerns; Weight/Shape Over-evaluation), as well as a total score calculated as the mean of all items. Possible values range from 0 to 6, with higher scores indicating greater eating disorder symptom severity. A recent validation study in a Mexican clinical sample supported its factorial structure, internal consistency, and convergent validity (32).

Secondary outcomes will address constructs consistent with sociocultural and cognitive-behavioral models of eating disorder risk and protection. BD will be assessed with the Body Shape Questionnaire-8 (BSQ-8; 33), with evidence of validity in Spanish-speaking populations (34). Scores range from 8 to 48, with higher values indicating greater dissatisfaction. Positive body image will be measured with the Body Appreciation Scale-2 (BAS-2; 23), which has demonstrated measurement invariance and reliability in Latin American adolescents (35); the BAS-2 consists of 10 items rated from 1 to 5, with higher scores reflecting greater body appreciation. Social physique anxiety will be assessed with the Social Physique Anxiety Scale-7 (SPAS-7; 22), supported by evidence of reliability and validity in Spanish-speaking samples (36); scores range from 1 to 5, with higher values indicating greater anxiety in social contexts. Internalization of sociocultural appearance ideals will be measured with the Thin-Ideal Internalization subscale of the Sociocultural Attitudes Towards Appearance Questionnaire-4 (SATAQ-4; (37), which has been culturally adapted and validated in Latin American populations (38). This subscale includes 5 items rated from 1 to 5, with higher scores indicating stronger thin-ideal internalization. Finally, appearance-related social comparisons will be evaluated with the Physical Appearance Comparison Scale (PACS; (39), with validation evidence in Spanish-speaking populations (40). Items are rated on a 5-point Likert scale from 0 (never) to 4 (always), and higher mean scores indicate more frequent appearance-based comparisons.

All self-report instruments will be administered at baseline (T1, week 0), post-intervention (T2, week 2), and 4-week follow-up (T3, week 6).

### Randomization, allocation, and blinding

Participants will be randomly assigned in a 1:1 ratio to the intervention (BIP) or waitlist control group using a computer-generated sequence with permuted blocks (undisclosed block sizes) created by a researcher not involved in recruitment or intervention delivery. Allocation will be concealed until assignment, after which participants will be informed of their group by the study coordinator via email.

This is an open-label trial; neither participants nor facilitators will be blinded to group assignment. Given the behavioral nature of the intervention, blinding of participants and facilitators was not feasible. To minimize bias, outcomes will be collected via standardized electronic self-report measures, and datasets provided to the analysis team will be de-identified.

### Sample size and statistical analysis

This pilot RCT aims to recruit 30 participants in total, with 15 allocated to the intervention arm and 15 to the waitlist control. Participants in the intervention condition will be organized into two groups of approximately 7–8 participants each, consistent with recommendations for small-group delivery of the BIP. This sample size is a pragmatic target aligned with recommendations for external pilots, where small samples are sufficient to assess feasibility and provide preliminary estimates for future trials (24, 27, 41). The study is therefore not powered for hypothesis testing; the focus is on feasibility and preliminary effect estimation.

Analyses will follow an intention-to-treat approach. All randomized participants will be invited to complete T2 and T3 assessments, including those who discontinue the intervention or withdraw from other study activities. Analyses will include all randomized participants under the ITT framework, with exploratory per-protocol analyses limited to participants who attend both sessions. Continuous outcomes (ED-15 and secondary measures) will be examined using linear mixed-effects models with Group, Time, and their interaction as fixed effects, and a random intercept for participants. Between-group differences in change from baseline to post-intervention and follow-up will be reported with 95% confidence intervals and standardized effect sizes. Missing data will be handled using maximum likelihood estimation within the mixed-effects models, which accommodate incomplete data under a missing-at-random (MAR) assumption. Feasibility outcomes (recruitment, retention, adherence, satisfaction) will be summarized descriptively with proportions and 95% CIs. All analyses will be conducted in R (R Core Team).

### Ethics, data management, and dissemination

This study was approved by the Ethics Committee of the Instituto Tecnológico de Monterrey (Nro.: CA-EMCS-015) and will be conducted in accordance with the Declaration of Helsinki (42) and local regulatory standards. All participants will provide written informed consent before enrollment.

Data will be collected using secure online survey platforms and stored in encrypted files accessible only to the research team. Identifiable information will be kept separate from study data to ensure confidentiality. After de-identification, the anonymized dataset and statistical code will be made publicly available in the Open Science Framework (https://osf.io), in line with open science practices.

The protocol has been prospectively registered at ClinicalTrials.gov (identifier: NCT07193043), ensuring transparency and adherence to SPIRIT 2013 guidelines (29). Findings will be disseminated through peer-reviewed publications, conference presentations, and summary reports to participating institutions.

## Discussion

This study protocol addresses a critical gap in ED prevention research by outlining the first randomized controlled evaluation of the BIP in Mexico. To our knowledge, no rigorously controlled randomized trials of culturally adapted ED prevention programs have been conducted in Mexico, making this protocol an important step toward expanding the evidence base beyond Western contexts. Despite the high prevalence of disordered eating behaviors and BD in Latin America, preventive interventions have rarely been subjected to rigorous trials in the region, with existing studies limited by methodological constraints such as the absence of control groups (20). Building on prior adaptations of the Body Project (21), this pilot RCT seeks to test the feasibility and acceptability of the BIP in a university population, thereby contributing to culturally relevant evidence and reducing reliance on Eurocentric data. By preregistering the trial and adhering to SPIRIT guidelines (29), the study also aims to advance transparency and reproducibility in the field.

This protocol has several strengths. It represents the first attempt to evaluate the BIP through a randomized controlled design in Mexico, addressing a major gap in the regional evidence base. Previous work has documented the adaptation and implementation of the program across Latin America, highlighting its feasibility and acceptability in diverse settings (21). Earlier research in Mexico also showed promising reductions in BD and thin-ideal internalization, although limited by the absence of a control group (20). Building on this groundwork, the current pilot RCT aims to provide a more rigorous test of feasibility, acceptability, and exploratory clinical outcomes. Additional strengths include the use of validated instruments adapted for Latin American populations (e.g., ED-15, BAS-2), adherence to SPIRIT guidelines, and prospective registration at ClinicalTrials.gov, all of which enhance methodological transparency and reproducibility. Such use of psychometrically validated measures is consistent with best practices in prevention research, which emphasize cultural adaptation and cross-cultural measurement invariance.

Several limitations should also be acknowledged. As a pilot RCT, the study is not powered to detect small-to-moderate effects, and findings regarding clinical outcomes must therefore be considered preliminary. The relatively small sample size, although consistent with recommendations for external pilots, may limit the generalizability of results and the stability of effect size estimates. In addition, the use of self-report measures, while efficient and validated in Mexican populations, introduces potential biases such as social desirability and recall effects. Finally, because the trial is restricted to female university students from a single region of Mexico, the results may not be representative of other demographic groups, such as men, non-binary individuals, or adolescents outside the university setting. Importantly, feasibility and acceptability—not efficacy—are the primary outcomes of this study, which should guide interpretation of future results. These limitations are common to external pilot trials, whose purpose is to establish feasibility and optimize trial procedures rather than provide definitive evidence of efficacy.

The present trial has important implications for both practice and future research. By systematically testing the feasibility and acceptability of the BIP in a Mexican university context, the study will generate insights into how dissonance-based prevention programs can be culturally adapted and delivered outside of high-income Western settings. Findings will inform whether the intervention retains its theoretical mechanisms, such as reducing thin-ideal internalization and appearance-based social comparisons, while also promoting positive indicators like body appreciation. These mechanisms are consistent with sociocultural and cognitive-behavioral models of ED pathology, which identify internalization of appearance ideals, social comparison, and body image overvaluation as central risk factors for disordered eating. Moreover, the pilot data will guide the design of a fully powered RCT, providing preliminary estimates of variability and highlighting potential challenges in recruitment, retention, or implementation.

Findings from this pilot will guide key decisions for the full RCT, including refinements to recruitment and session procedures, identification of primary and secondary outcomes, and sample-size estimation based on variability and attrition patterns. These indicators will define the final protocol for the definitive trial.

In addition to informing the design of a larger trial, this study may also contribute to broader discussions on public health priorities in Latin America. Given the clinical and economic burden associated with EDs, generating evidence on the feasibility of culturally adapted prevention programs can provide a foundation for scalable interventions. Such data are essential for guiding policy decisions and resource allocation in contexts where preventive strategies remain scarce and health systems face growing demands.

Beyond its methodological contribution, this protocol aligns with efforts to address inequities in global mental health research by expanding the evidence base for ED prevention in underrepresented regions. By integrating principles of open science and preregistration, this pilot RCT not only contributes to more transparent and culturally inclusive research practices but also represents a critical step toward establishing scalable eating disorder prevention strategies in Latin America. While a small number of trials have been conducted in Brazil (43, 44), the evidence base in the region remains scarce and methodologically limited. Future trials should examine whether findings generalize to other Latin American populations and to diverse educational or community settings. Such efforts will be essential to determine scalability and transferability across low and middle-income country contexts (45). Importantly, this study may also serve as a model for developing and testing culturally adapted prevention programs for eating disorders in other underrepresented regions worldwide, thereby contributing to a more equitable and globally relevant evidence base.
